# “Gotta have balls”: bovine testicles and video magnification imaging in the varicocelectomy training

**DOI:** 10.1590/acb405225

**Published:** 2025-07-07

**Authors:** Victor Matheus Mendonça de Araújo, Manuela Rodrigues Neiva Fernandes, Lívia Guerreiro de Barros Bentes, Rafael Silva Lemos, Herick Pampolha Huet de Bacelar, Luis Otávio Amaral Duarte Pinto

**Affiliations:** 1 Universidade do Estado do Pará – Faculdade de Medicina – Laboratório de Cirurgia Experimental – Belém (PA) – Brasil.

**Keywords:** Simulation Training, Microsurgery, Varicocele, Urological Surgical Procedures

## Abstract

**Purpose:**

To describe the varicocelectomy model using bull testicles and to evaluate microsurgical practice using the surgical microscope and video magnification system.

**Methods:**

Bovine testicles and spermatic cords were used, with the medial portion of the cord left free for the microsurgical varicocelectomy technique. Twenty 3rd-year medical students were divided into two groups, the video magnification system group (VSMG) and the surgical microscope group (SMG), to simulate varicocelectomy in the proposed model. Five training sessions were carried out for both groups (D1, D7, D14, D21, and D28), as well as a reassessment (D63), with a checklist applied on the first, third, and fifth day of training and on reassessment.

**Results:**

The model provides practical support for training in instrument handling, dissection of structures, anatomical identification, and vessel ligation as an alternative to learning the surgical technique. There was a drop in training time as the weeks went by, with no significant difference between the groups. There was no statistical difference in time (D14, D28, D63) or scores between groups.

**Conclusion:**

Microsurgical varicocelectomy training using testicles and sperm cords of bovine origin in the video magnification system and surgical microscope contributed to the acquisition of skills.

## Introduction

The training of a surgeon involves the development of cognitive skills, motor techniques, and surgical practice^1^. In urology, the variety of diagnostic and therapies are large, mainly involving specialized surgical procedures, such as robotic surgery, laser technology, and microsurgery^2^^,^ 3. Currently, this surgical learning uses simulators developed to train students, residents and urologists, aiming to overcome the “see one, do one” practice, and seeking to increase the complexity of training, with reduced time and patient safety^1^^,^ 4. Microsurgery is one of these fundamental techniques to be trained, especially in urology, due to the need for precision in anatomical structures, as occurs in the correction of varicocele^5^.

Microsurgical sub-inguinal varicocelectomy is the name of the technique considered the gold standard for the treatment of varicocele^6^. Many urologists do not have adequate microsurgical skills to perform this procedure^7^^–^ 9. Despite the Brazilian Ministry of Education, with the medical residency program in urology, it is a mandatory competence to master the technique for surgical correction of varicocele at the end of the first year^10^. Furthermore, the Accreditation Council of Graduate Medical Education does not require microsurgical training for urology residents in the United States of America, a factor that may be related to skills deficiency in this area^8^. Another important factor is the contact with this pathology and others of a benign nature, which can be deficient in several specialization centers^11^. Therefore, training microsurgical management of these professionals is essential to ensure patient safety and the expected result of the surgical procedure.

To address this lack of skills, some training models were created^12^^,^ 13. However, given the Russel-Burch principle, which establishes the premises of reducing, refining and replacing the use of animals in research, the development of non-living models–which tend to have a lower assembly cost–has been essential for the dissemination of simulation models in microsurgical practice around the world14. This promotes the accessibility of simulators with skill acquisition, in addition to encouraging new projects and simulation-based education programs such as three-dimensional printers, video games, virtual reality, or even the use of discarded animal tissues^14^^,^ 15.

It should be noted that the use of simulators provides a controlled environment that does not involve the risks of intraoperative complications for patients, which allows for gradual learning that follows a sequential plan that increases the difficulty of execution progressively^4^^,^ 16. Therefore, specializations and residency programs include training models, mainly anatomical parts of *ex vivo* animals, in continuing education^17^, making patient safe and learning a priority for specialists.

Therefore, the objective of this study was to describe a new model for the acquisition of microsurgical skills of varicocelectomy from bovine testicles, to validate this model through practice with medical students and compare the differences in training on a video magnification system and a surgical microscope.

## Methods

This study is presented as experimental and longitudinal, having been carried out by the Brazilian law regulating the scientific use of animals (Law No. 11,794/08). It was approved by the Research Ethics Committee (No. 58872022.4.0000.5174) and was granted exemption from the Research Ethics Committee on Animal Use of the institution where the study was developed, on the premises of the Experimental Surgery Laboratory located at the Center for Biological and Health Sciences, at the Universidade Estadual do Pará (UEPA).

### Model description

**Figure 1:**
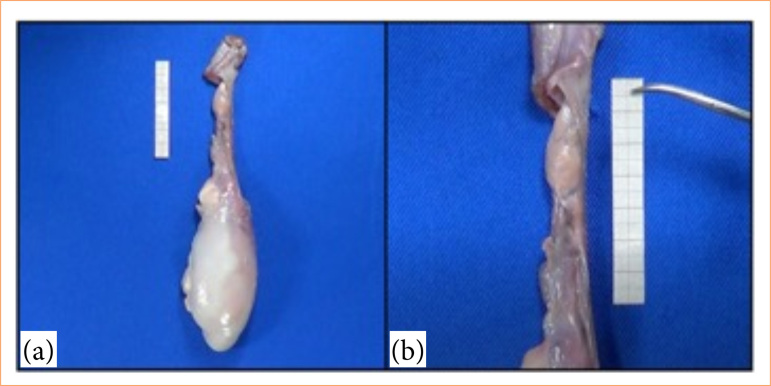
Anatomical view of a bovine testicle and spermatic cord prior to surgical intervention. **(a)** Bovine testicles and spermatic cord. **(b)** Medial portion of the spermatic cord.

Source: Elaborated by the authors.

In the microsurgical varicocelectomy training model, bovine testicles and spermatic cords were acquired at a local breeding site of commercialization of cattle meat, after castration. The material was stored under refrigeration at 5°C and then exposed to room temperature (27°C) 1 hour before the procedure started. The testicle and spermatic cord were previously washed for better visualization of the structures, with subsequent fixation on a flat surface. Adhesive tapes were used in three different locations for fixation: testicular surface, distal and proximal portions of the spermatic cord. The medial portion of the cord, measuring approximately 5 cm, was left free for the microsurgical varicocelectomy technique (Fig. 1).

### Assessment of model feasibility

Initially, the model was considered due to the anatomical similarity with the testicle human. From there, the bovine testicles were tested by a urologist linked to the UEPA, which has more than 10 years of experience in microsurgery and infertility treatment, as well as that is a preceptor of medical residency in Urology. The test consisted of dissecting the cremaster muscle and fascia sperm until reaching the chosen vein, vein isolation, two-part ligation venous veins with 4-0 silk thread, and cutting at the midpoint of the ligature using straight Iris scissors 11 cm.

After the test, the urologist was asked about the model and its academic applicability according to the following questions:

- Can the model simulate the characteristics of the human tissue?;
- Is the use of the model tangible for medical students?;
- Does the model allow the gain of skills in varicocelectomy?

### Experimental groups

An inclusion criterion for participation in the research was to be a third-year student of Medicine at the UEPA who have already taken the Surgical Skills discipline. Those who did not agree to sign the Free and Informed Term of Agreement or that would not be available on training days and evaluations were excluded from the research. In the end, 20 students met the proposed criteria, which were divided into two groups, each containing 10 people.

- Video magnification system group (VSMG): 10 participants were selected randomly to compose the group, which trained microsurgery in a low-cost model of varicocelectomy under the video magnification system^14^^,^ 18 installed in the laboratory of UEPA Experimental Surgery and composed of a Sony^©^ Handycam HDR-XR160 camera connected to a 55’ Curl Full HD TV via an HDMI cable. The participants were evaluated under a microscope surgical;
- Surgical microscope group (SMG): 10 participants were randomly selected to compose the group, which trained microsurgery in a varicocelectomy training model under a conventional surgical microscope DF Vasconcelos 9000, also being evaluated under the microscope.

### Operative technique

The technique used in the microsurgical varicocelectomy training model corresponds to the following steps: dissection of the cremaster muscle and spermatic fascia until the reach of the chosen vein; vein isolation; ligation of two venous portions with silk thread 4-0; and cutting at the midpoint of the ligatures by straight 11-cm Iris scissors.

### Training and assessment

There were five training sessions for both groups (D1, D7, D14, D21, and D28), with one training per week, and finally a reassessment nine weeks after the start of the training (D63). On the first, third and fifth day of training, and on reassessment (D1, D14, D28 and D63), the Campos et al. checklist of 2020^7^ for varicocelectomy microsurgery was applied, assessing skills through the following questions:

- Does the participant correctly adjust, position, and handle instruments and the microscope, maintaining the sterile field?;
- Does the participant recognize and adequately dissect adjacent structures?;
- Does the participant adequately dissect the veins, differentiating them from arteries and lymphatic vessels?;
- Does the participant adequately ligate and section the spermatic vein?

Each positive answer question was equivalent to 1 point. Otherwise, the point was 0. In addition, the time spent training each week was measured. Interns with more than three years of experience in microsurgery were responsible for the participants’ evaluation.

### Statistical analysis

Microsoft Word and Excel softwares were used to tabulate data and create graphs and figures. The BioEstat 5.4 software was used to carry out the statistical analysis. For normally distributed data, the analysis of variance (ANOVA) parametric test was used, and for those with an abnormal distribution the non-parametric Kruskal-Wallis test. Finally, for comparison, paired t-Student was used. Statistical significance was set at *p* ≤ 0.05.

## Results

**Figure 2:**
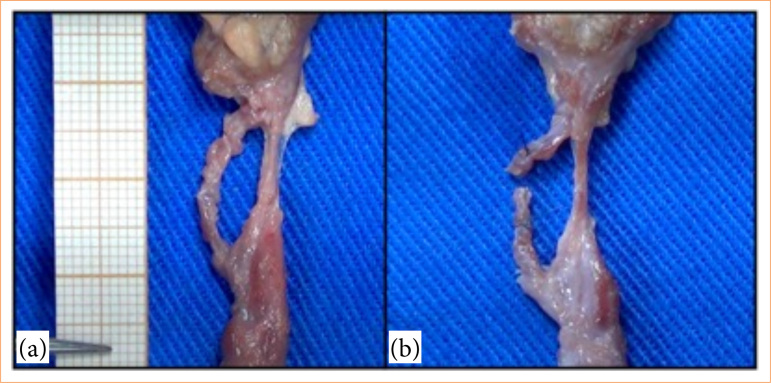
Dissected spermatic cord of a bovine specimen showing veins of the pampiniform plexus before surgical ligation. **(a)** Isolated vein. **(b)** Vein divided after ligation.

Source: Elaborated by the authors.

The varicocelectomy model provides practical support for training handling instruments, dissection of structures, anatomical identification, and ligation of vessels. The urologist’s assessment considered the model suitable for learning skills in microsurgery and varicocelectomy technique, being pertinent in the stage of dissection of the planes, in which it occurred similarly (Fig. 2). Furthermore, animal tissue can represent closely the human testicle, both in texture and size, and is suitable for undergraduate medical students.

The material used to create the training models was free of charge since the bovine testicles used would be discarded after the castration procedure in local breeding facilities. The values relating to the materials used during the training sessions can be found in Table 1.

**Table 1:** Total training costs.

Material	Price (unit)	Price (total)
4-0 silk thread	BRL 5,37	BRL 912,90
Bovine testicles	Not applicable	Not applicable

Source: Elaborated by the authors

**Figure 3:**
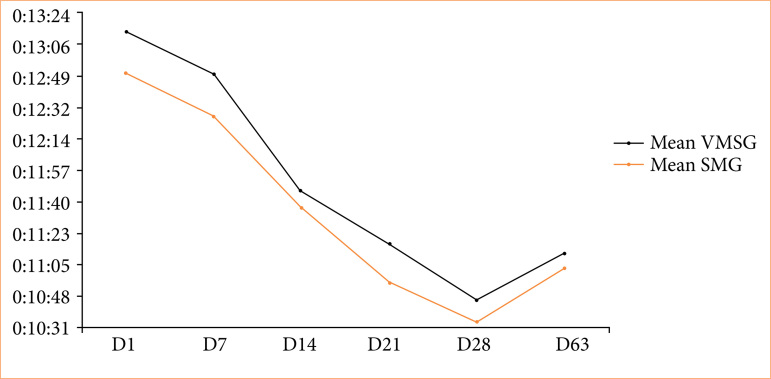
Average training time in minutes and seconds in the model varicocelectomy per week.

VMSG: video magnification system group; SMG: surgical microscope group. Source: Elaborated by the authors.

Time analysis in this training model demonstrated a drop in time carrying out the procedure over the weeks (Fig. 3). The average time of D1 and D28 obtained a significant statistical difference (*p* < 0.05), similar to D1 and D63 (*p* < 0.05). There was also a distinction between the times of both groups on D28 and D63 (*p* < 0.05), five weeks apart. In comparison between the groups, the SMG obtained the lowest time throughout the period. There was no statistical difference between the groups in days D14, D28 and D63 (Table 2).

**Table 2:** Comparison between groups of average time, in minutes and seconds, and standard deviation of training in the varicocelectomy model per day.

Training	VSMG	SMG	P-value
D1	13:15 ± 00:26	12:49 ± 00:17	0.05
D7	12:49 ± 00:17	12:23 ± 00:26	0.01
D14	11:48 ± 00:26	11:40 ± 00:09	0.39
D21	11:14 ± 00:26	10:57 ± 00:17	0.03
D28	10:48 ± 00:17	10:31 ± 00:17	0.08
D63	11:14 ± 00:17	11:05 ± 00:17	0.35

VMSG: video magnification system group; SMG: surgical microscope group. Source: Elaborated by the authors.

**Figure 4:**
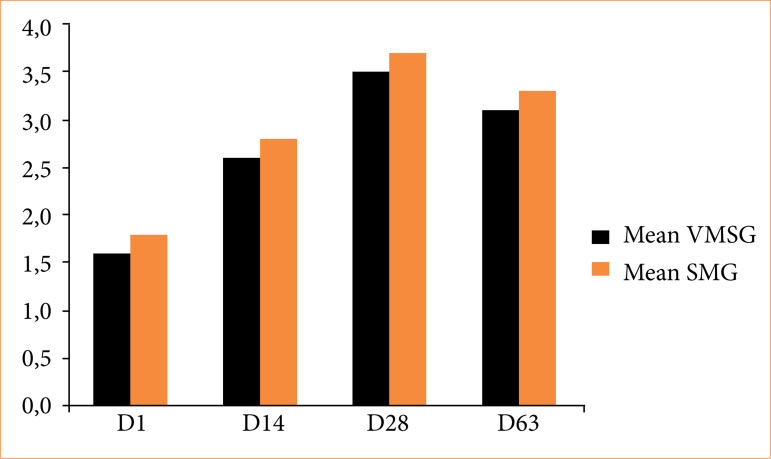
Average checklist score of the training groups in the varicocelectomy model per day.

VMSG: video magnification system group; SMG: surgical microscope group. Source: Elaborated by the authors.

The average training checklist score increased as the days went by (Fig. 4). Comparing D1 and D28, there was a significant statistical difference (*p* = 0.05), similar to the comparison of D14 and D28 (*p* < 0.05). The reevaluation (D63) made a significant difference when compared to the first day (D1) (p < 0.01), but did not demonstrate a difference when compared with D14 and D28 (*p* > 0.05). Between the groups, there was no statistically significant difference on any days evaluated (Table 3).

**Table 3:** Comparison per day of the mean score and standard deviation in the checklist of the groups in the training in the varicocelectomy model.

Training	VSMG	SMG	P-value
D1	1.6 ± 0.699	1.8 ± 0.632	0.25
D14	2.6 ± 0.516	2.8 ± 0.421	0.17
D28	3.5 ± 0.707	3.7 ± 0.483	0.23
D63	3.1 ± 0.316	3.3 ± 0.674	0.20

VMSG: video magnification system group; SMG: surgical microscope group. Source: Elaborated by the authors.

## Discussion

The accessibility of magnification technologies and training models frequently offers opportunities to continue the delicate motor practice and progression of microsurgical skills. In this context, with simulation tools for urological microsurgery, given its application in the varicocele correction technique, the apprentices can develop dissection skills, differentiation of structures, ligation, and section, in order to reproduce the surgical scenario without compromising the clinical status of patients^7^^,^ 19.

Regarding models in urological microsurgical training, it is not uncommon the use of simulators for practicing vasectomy reversal, such as simulators made with a three-dimensional printer, or even using pig testicles^20^^,^ 21. However, no other models were found in the literature that used testicles and spermatic cord non-humans for urological training in varicocelectomy. Taking into account other non-living animal tissues, only the model described by Campos et al.^7^ was found, which uses the human placenta.

In this research, the choice to use bovine testicles was due to their anatomical similarity to human tissue^22^. The training model used, in addition to innovation and to allow the reproduction of the difficulties and anatomical stages of surgery in humans, still conquers the concept of relative replacement, by the principle of 3Rs (reduction, refinement, and replacement)^23^ in animal research, when reassigning tissue cattle that would be neglected. From this perspective, it was possible that the cost for the development of this work only included the threads used in training and assessment sessions.

Regarding the equipment used in training between the different groups of this study, the video magnification can increase accessibility to microsurgical learning, as it enables high-definition images even in centers that do not have high technology, such as the UEPA Experimental Surgery Laboratory, in addition to being capable of reproducibility^18^^,^ 23 ^,^ 24. An important area of analysis regarding the microsurgical visual augmentation device is the time required to perform the varicocelectomy procedure. Throughout the evaluation weeks, both the group that trained on the video magnification system and the group that trained on the surgical microscope showed significant reductions when comparing the first day of quantification (D1) with the last day of training (D28), or even with the day reevaluation five weeks after the completion of training (D63). This situation corroborates the understanding that microsurgical learning is positively influenced by interval training since cognitive preparation and mental rehearsal between sessions deeply systematize the specific memory skill^20^^,^ 25 ^,^ 26.

When specifically comparing the times of the D28 with those of the D63 in both groups, a statistically significant increase can be seen during the five-week evaluation after completion of training. Similarly, there was a drop in the checklist score in the D63 concerning D28, although the grade on the last day of assessment is still higher than those obtained in D1 and D14. It is well established that regular practice has a relevant influence on the progression of manual learning in microsurgery^7^^,^ 27, but there are disagreements in the literature regarding the precise cut-off time for forgetting. Some studies obtained similar results, with a drop in performance in the evaluation after four weeks^25^, while other studies, which evaluate more experienced professionals, such as resident doctors, noted a longer-lasting performance^27^^,^ 28.

The advantages of using the video system for surgical magnification in relation to the microscope refer to better ergonomics, easy recording, and high-definition images^18^. However, its main limitation concerns the lack of stereoscopic view, which makes it difficult to precise the three-dimensional distance of objects in the field^24^. This aspect may have contributed to the groups that trained on the video magnification system obtained longer times in all assessments, with this difference being significant on half of the days in which the performance of varicocelectomy was timed.

Measuring acquired skills is essential to indicate the effectiveness of an educational process^28^. Therefore, it was decided to use in this study the checklist validated by Campos et al.^7^ to evaluate the gain of skills in varicocelectomy training in simulation models. We chose to use the checklist instead of more complex scales of performance quantification because it was noted that it was the only validated artifact specifically for the analysis of varicocelectomy practice. The authors hope to give continuity to this thematic approach while using scales developed for the microsurgical training assessment, such as the Objective Structured Assessment of Technical Skill^29^, which perhaps provides a more detailed assessment^28^.

Regarding the evaluation of student performance, when comparing the average scores on the checklist, in both groups, there was a progressive increase in the score, from the first to the last day of training, highlighting the role of repetition practice in gaining urological microsurgical skills, especially when sessions were held^20^^,^ 21.

Furthermore, no significant statistical difference was found between the scores from the group that trained in the video magnification system and those from the surgical microscope group, indicating that initial training in an alternative imaging tool, even if lacking visibility in three dimensions, provides the evolution of skills associated with handling, dissection, ligation and section, even when the assessment is made in a more reliable tool for the experience of delicate urological procedures, using the surgical microscope^18^^,^ 25 ^,^ 28.

One of the limitations of this study was associated with the absence of blood in the sperm cord veins, leaving them withered and dry, which made it more challenging to perform the ligation, increasing the chances of rupture. Another obstacle encountered was the need to store bovine testicles in a freezer for more than 60 days, which potentially caused damage to the texture of the tissue and the venous structure of the sperm cord, factors that may have interfered with the quality of training.

Finally, two suggestions are made for future publications that address training of varicocelectomy in animal models: the use of contrast liquids that can simulate blood, and the use of testicles obtained immediately after castration, facilitating the handling of these structures and improving the technique for making ligatures. Further studies involving not only medical students but also urologists and residents in Urology are also suggested, for which microsurgical technical improvement in varicocele surgery would be of great benefit.

## Conclusion

It was possible to verify that varicocelectomy microsurgical training using testicles and spermatic cords of bovine origin in a video magnification system contributed to obtaining skills for the adequate performance of this procedure urology under the surgical microscope.

## Data Availability

All data sets were generated or analyzed in the current study.
